# Computer Program for Detection and Analyzing the Porin-Mediated Antibiotic Resistance of Bacteria

**DOI:** 10.17691/stm2021.13.6.02

**Published:** 2021-12-28

**Authors:** T.A. Savinova, A.A. Samchenko, Y.A. Bocharova, N.A. Mayansky, I.V. Chebotar

**Affiliations:** Leading Researcher, Laboratory of Molecular Microbiology; Pirogov National Research Medical University, 1 Ostrovityanova St., Moscow, 117997, Russia;; Researcher, Laboratory of Structure and Dynamics of Biomolecular Systems; Institute of Cell Biophysics of the Russian Academy of Sciences — Subdivision of the Federal Research Center “Pushchino Scientific Center for Biological Research of the Russian Academy of Sciences”, 3 Institutskaya St., Moscow Region, Pushchino, 142290, Russia; Senior Researcher, Laboratory of Molecular Microbiology; Pirogov National Research Medical University, 1 Ostrovityanova St., Moscow, 117997, Russia;; Professor of the Russian Academy of Sciences, Head of the Center for Laboratory Diagnostics, Russian Children’s Clinical Hospital; Pirogov National Research Medical University, 1 Ostrovityanova St., Moscow, 117997, Russia;; Head of the Laboratory of Molecular Microbiology; Pirogov National Research Medical University, 1 Ostrovityanova St., Moscow, 117997, Russia;

**Keywords:** antibiotic resistance, *Pseudomonas aeruginosa*, ОpdP, ОprD, ОpdD, porin genes, carbapenems

## Abstract

**The aim of this work** was to develop a new software tool for identifying gene mutations that determine the porin-mediated resistance to antibiotics in gram-negative bacteria and to demonstrate the functionality of this program by detecting porin-mediated resistance to carbapenems in clinical isolates of *Pseudomonas aeruginosa*.

**Materials and Methods:**

The proposed algorithm is based on searching for a correspondence between the reference and the studied genes. When the sought nucleotide sequence is found in the analyzed genome, it is compared with the reference one and analyzed. The genomic analysis is then verified by comparing between the amino acid sequences encoded by the reference and studied genes. The genes of the susceptible *P. aeruginosa* ATCC 27853 strain were used as the reference nucleotide sequences encoding for porins (OprD, OpdD, and OpdP) involved in the transport of carbapenems into the bacterial cell. The complete genomes of clinical *P. aeruginosa* isolates from the PATRIC database 3.6.9 and our own collection were used to test the functionality of the proposed program. The analyzed isolates were phenotypically characterized according to the CLSI standard. The search for carbapenemase genes in the studied genomes of *P. aeruginosa* was carried out using the ResFinder 4.1.

**Results:**

The developed program for detecting the genetic determinants of non-plasmid antibiotic resistance made it possible to identify mutations of various types and significance in the porin genes of *P. aeruginosa* clinical isolates. These mutations led to modifications of the peptide structure of porin proteins. Single amino acid substitutions prevailed in the OpdD and OpdP porins of carbapenem-susceptible and carbapenem-resistant isolates. In the carbapenem-resistant strains, the gene encoding for OprD porin was found heavily modified, including insertions and/or deletions, which led to premature termination of porin synthesis. In several isolates resistant to meropenem, no mutations were detected in the gene encoding for OprD, which might be associated with alternative mechanisms of resistance to carbapenems.

**Conclusion:**

The proposed software product can become an effective tool for deciphering the molecular genetic mechanisms of bacterial chromosomal resistance to antibiotics. Testing the program revealed differences between the occurrences of mutations significant for carbapenem resistance in the *oprD*, *opdD*, and *opdP* genes.

## Introduction

The global occurrence of antimicrobial resistance (AMR), has become one of the most important problems of modern health care [1]. The situation with AMR is expected to get worse due to the large-scale use of antibiotics associated with the COVID-19 pandemic [2, 3]. It is for medical science to improve methods for assessing antibiotic resistance and deciphering its mechanisms. At the present stage, the commonly used phenotypic characterization of AMR is insufficient for successfully fighting the AMR; the phenotypic profile of resistant bacteria should be supplemented by an insight into the mechanisms of its formation [4].

Methods for identifying genetic determinants of resistance are among the most informative methods for describing the mechanisms of AMR formation. The main determinants of AMR are traditionally subdivided into plasmid and chromosomal ones. Plasmid resistance genes are transmittable from cell to cell in a horizontal way; they can be localized both in the plasmid and in the chromosome [5–7]. Chromosomal resistance is associated with mutations (point mutations, small insertions/deletions, extensive insertions/deletions, including insertions of mobile genetic elements) in chromosomal genes specific for this type of bacteria [8–10].

To search for plasmid genes involved in AMR, online software tools/programs that require a locally installed (standalone) version have been created and are successfully used. These include ResFinder, CARD, ARDB, ARG-ANNOT, and other resources [11–14]. They are primarily aimed at finding genes that are transmitted as mobile genetic elements, for example, genes for beta-lactamases (resistance to beta-lactam antibiotics), aminoglycoside transferases (resistance to aminoglycosides and fluoroquinolones), chloramphenicol acetyltransferases (resistance to chloramphenicol), glutathione-S-transferases (resistance to fosfomycin), genes for ribosomal protection proteins (resistance to tetracycline) and other determinants of antibiotic resistance. Some of the existing tools are capable of searching for chromosomal determinants of drug resistance as well (PointFinder, CARD).

Unfortunately, the functionality of these software tools does not always allow one to identify and analyze important genetic determinants of AMR associated with changes in porins responsible for transportation of antibiotics into the cell, regulatory genes for global efflux systems, and genes encoding for antibiotic targets. In particular, this applies to deciphering the molecular mechanisms of resistance to latest generation of antibiotics including carbapenems.

**The aim of this work** was to develop a new software tool for identifying gene mutations that determine the porin-dependent resistance to antibiotics in gram-negative bacteria and to demonstrate the functionality of this program by detecting porin-mediated resistance to carbapenems in clinical isolates of *P. aeruginosa*.

## Materials and Methods

The overall study design included:

1. creating a database of porins’ reference genes involved in the antibiotic transport through the outer bacterial membrane into the periplasmic space;
2. obtaining the amino acid sequences of porins in the given clinical isolates (based on their full genomic data);
3. using the originally developed software to identify the genetic determinants of non-plasmid antibiotic resistance [15]; the algorithm must be able to detect the differences between the analyzed gene (or protein) sequence and the reference sequences stored in the database; testing the program using the model of porin-mediated bacterial resistance to meropenem and imipenem.

The program includes a database of reference porin genes involved in the AMR development in clinically significant species of gram-negative bacteria, including *Pseudomonas aeruginosa*, *Klebsiella pneumoniae*, *Escherichia coli*, *Enterobacter spp.*, *Salmonella enterica*, etc. The sequences of the reference genes were obtained from the NCBI database (https://www.ncbi.nlm.nih.gov/nuccore/) by selecting the sequences of porin genes in strains that are recommended by the international standards EUCAST (European Committee on Antimicrobial Susceptibility Testing) and CLSI (Clinical and Laboratory Standards Institute, USA) as antibiotic susceptibility standards. In the present study, for the reference, we used the drug-susceptible ATCC 27853 strain of *P. aeruginosa* and the nucleotide sequences of the genes encoding for the respective porins OprD/OccD1, OpdD/OccK7, and OpdP/OccD3, whose participation in the mechanism of resistance to carbapenems was confirmed earlier (Table 1).

**Table 1:** *Pseudomonas aeruginosa* porins involved in the transport of carbapenems

Porin name	Alternative name	Substrate	Literature
OprD	OccD1	Meropenem, imipenem, lysine, histidine, arginine, ornithine	[16–18]
OpdD	OccK7	Meropenem	[18]
OpdP	OccD3	Meropenem, glycine-glutamate, arginine	[19, 20]

As a negative control (strains lacking species-specific *P. aeruginosa* porins), 10 genomes from the PATRIC 3.6.9 database (https://www.patricbrc.org/) of five different species were analyzed: *Acinetobacter baumannii* (SP5515, GML-KP48-AB-TR), *Klebsiella pneumoniae* (AR0361, AR438), *Escherichia coli* (AR435, AR0450), *Staphylococcus aureus* (AR464, AR0216), and *Enterococcus faecium* (VREN1530, VREN2775).

To test the capabilities of the proposed program, complete genomes (complete assemblies or contigs) of *P. aeruginosa* from two sources were used. The first source was the PATRIC 3.6.9 database, from which 15 genomes of clinically relevant *P. aeruginosa* strains were obtained together with the AMR characteristics of these isolates. The second source of genomes (10 samples) was our own collection of DNA from clinically-relevant strains, which had their AMR spectra (sensitivity to meropenem and imipinem) previously characterized by the phenotypic CLSI standard, 2020 (https://clsi.org/standards/).

The search for adaptive genes of carbapenem resistance (genes of carbapenemases) in the studied genomes of *P. aeruginosa* was carried out using the ResFinder 4.1 resource (https://cge.cbs.dtu.dk/services/ResFinder/).

The function of the program is based on the algorithm depicted in the Figure. At the first stage, a search for a match between the reference and studied genes is performed using the BLAST program based on the best-matching choice [21]. When the desired nucleotide sequence is found in the analyzed genome, it is compared to the reference one. If they differ, the data is analyzed by the program and the result is automatically displayed in a text or tabular format to present the detected changes in the nucleotide sequence of the gene. The significance of the detected changes for the emergence of AMR is tested by analyzing the synonymy of substitutions in the amino acid sequence of porin. Synonymy analysis is performed automatically; this function is incorporated in the program code.

**Figure:**
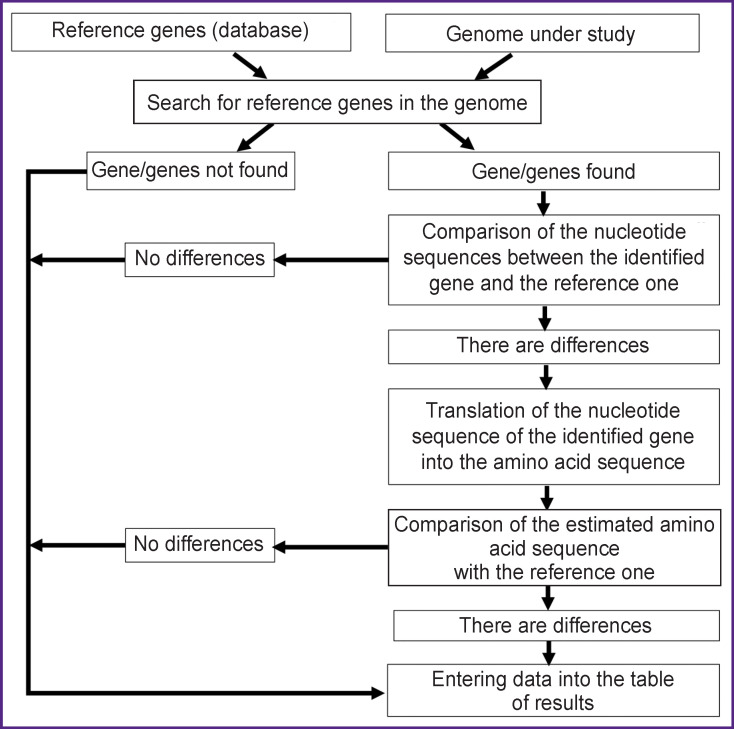
Algorithm of the computer program for the detection and analysis of porin-mediated antimicrobial resistance in gram-negative bacteria

## Results

Genes encoding for the OprD, OpdD, and OpdP porins were found in the genomes of all *P. aeruginosa* isolates (n=25) analyzed in this study. By contrast, none of these genes were found in the genomes of non-*P. aeruginosa* isolates (n=10) used as a negative control.

In the genomes of the 25 studied isolates of *P. aeruginosa*, mutations of various types and significance were identified. In 15 isolates, mutations of the *oprD* gene were detected, in 22 isolates — mutations of the *opdD* gene, and in 13 isolates — mutations of the *opdP* gene (Table 2). In total, 4 groups of mutations were found: those led to modifications of the porin peptide structure.

**Table 2:** Peptide structure modifications in porins of *Pseudomonas aeruginosa* genomes (n=25)

Modifications	The number of genomes in which mutations corresponding to porin changes were found		
	OprD	OpdD	OpdP
No changes in the product (genes are identical to the reference, or synonymous mutations in the gene)	10	3	12
AAS	3	18	12
Stop / AAS + stop	1	—	—
Frameshift + stop / AAS + frameshift + stop	5	4	1
AAS + INS	6/25	—	—

Here: AAS — amino acid substitution; stop — stop codon leading to premature stopping of porin synthesis; frameshift — shift of the reading frame; INS — amino acid insertion without frameshift.

Amino acid substitutions in the OpdD and OpdP porins were detected most often. Porins OprD and OpdD were characterized by a greater variety of modifications, including insertions and/or deletions accompanied by the appearance of a stop codon.

We then analyzed whether the phenotypic manifestation of AMR correlated with the presence of mutations in the porin genes or the presence of carbapenemases genes (genotype/phenotype ratio). The AMR was assessed by the data on participation of porins in the antibiotics transport through the outer membrane (see Table 1; Table 3). In 7 out of 8 isolates sensitive to meropenem, either no mutations were observed in the genes for the OprD and wild-type OpdP porins, or 1 to 4 mutations were detected in these genes. One meropenem susceptible isolate had an OprD mutant porin (16 amino acid substitutions). The most frequent *oprD* mutations in isolates resistant or with intermediate resistance to carbapenems were mutations that led to amino acid substitutions and insertion of two amino acids (6/22), as well as a frameshift mutation leading to premature termination of porin synthesis (5/22). In 3 isolates insensitive to meropenem, no mutations in *oprD* were detected.

**Table 3:** Mutations in *Pseudomonas aeruginosa* porin genes and phenotypic manifestations of antimicrobial resistance (n=25)

Isolate name	Resistance phenotype (CLSI)		Molecular structure of porin			Correspondence of phenotype and genotype	Notes
	Meropenem	Imipenem	OprD (meropenem, imipenem)	OpdD (meropenem)	OpdP (meropenem)		
PATRIC_JVIRSN8915	R	R	N43D E57S R59S V127L E185Q P186G V189T S267A T276A G310E G312R A315G L347M L359V K387S, frameshift and L432stop	WT	P31S	Yes	Gene *bla*_OXA-395_ found
PATRIC_MRSN994	R	R	N107R, frameshift and D113stop	WT	WT	Yes	Gene *bla*_OXA-396_ found
PATRIC_JVIRSN8139	I	R	N43D V127L E185Q, frameshift and D231stop	A10VS132T T247AD401N	A29EA424T P445T	Yes	
PATRIC_JVIRSN8912	R	R	N43D E57S R59S T103S K115T Q142stop	T247A A304G	A29EI159L	Yes	
PATRIC_MRSN4841	I	S	N43D E57S R59S T103S K115T F170L E185Q P186G V189T Q202E A210I K230E T240S T262N S267A G281A Q296K E301Q G310E A315G L359V V372M insS S374N S375N S376V S377G A379K G380N L381Y insG G423A	F19S L72S, frameshift and L117stop	A29E T30S	Not obvious	Gene *bla*_OXA-488_ found
PATRIC_MRSN443463	S	S	WT	A183T P245Q T247A	WT	Yes	
PATRIC_MRSN9718	R	S	WT	T247A R268H	A29E	Yes	Gene *bla*_OXA-396_ found
PATRIC_MRSN435288	S	I	WT	V77I	WT	Yes	Gene *bla*_OXA-50_ found
PATRIC_MRSN321	R	R	S278P	S132T T247A A301V	WT	Yes	Gene *bla*_OXA-396_ found
PATRIC_MRSN25678	I	S	N43D E57S R59S V127L	S132T D163G N182H V240M L246F T247A D249G S298T	L15M N215T	Yes	
PATRIC_MRSN373401	I	R	WT	V77I	WT	Yes	Gene *bla*_OXA-486_ found
PATRIC_MRSN29192	R	I	N43D E57S R59S Q202E A210I K230E T240S T262N S267A G281A Q296K E301Q G310S L359V V372M insS S374N S375N S376V S377G A379K G380N L381Y insG	V77I T247A	WT	Yes	Genes *bla*_OXA-485_, *bla*_OXA-488_ found
PATRIC_MRSN409937	R	S	N43D E57S R59S Q202E A210I K230E T240S T262N S267A G281A Q296K E301Q G310R L359V V372M insS S374N S375N S376V S377G A379K G380N L381Y insG	WT	WT	Yes	Gene *bla*_OXA-50_ found
PATRIC_MRSN358800	R	R	N43D E57S R59S T103S K115T M141A, frameshift and D218stop	D231G T247A	A29E, frameshift and M81 stop	Yes	
PATRIC_MRSN401528	S	S	WT	V77I H110Y	WT	Yes	
Pa J 35-3	S	R	WT	V77I	WT	Yes	Gene *bla*_OXA-486_ found
Pa_212-2	S	R	N43D E57S R59S V127L E185Q P186G V189T S267A T276A G310E G312R A315G L347M L359V S401AQ422E	V77G	WT	Yes	
Pa_224-1	I	R	WT	A183T P245Q T247A	WT	Yes	Gene *bla*_OXA-50_ found
Pa_3-2	S	S	WT	V77I P202S T247A A304G	WT	Yes	
Pa_316-2	I	R	N43D E57S R59S T103S K115T F170L E185Q P186G V189T Q202E A210I K230E T240S T262N S267A G281A Q296K E301Q G310E A315G L359V V372M insS S374N S375N S376V S377G A379K G380N L381Y insG G423A	F19S L72S, frameshift and L117stop	A29E T30S	Yes	Gene *bla*_VIM-2_ found
Pa J 24-1	S	R	WT	V77IS114N R173S V377M	N103K	Yes	Gene *bla*_OXA-494_ found
Pa J 24-2	R	R	Frameshift S153P and G193stop	V77IS114N R173S V377M	N103K	Yes	Gene *bla*_OXA-396_ found
Pa_41728-1	S	R	WT	T247A T344I	N345D	Yes	Gene *bla*_OXA-488 _ found
Pa_41748-3	R	R	N43D E57S R59S T103S K115T F170L E185Q P186G V189T Q202E A210I K230E T240S T262N S267A G281A Q296K E301Q G310E A315G L359V V372M insS S374N S375N S376V S377G A379K G380N L381Y insG G423A	F19S L72S, frameshift and L117stop	A29E T30S	Yes	Gene *bla*_VIM-2_ found
Pa_98-3	R	R	N43D E57S R59S T103S K115T F170L E185Q P186G V189T Q202E A210I K230E T240S T262N S267A G281A Q296K E301Q G310E A315G L359V V372M insS S374N S375N S376V S377G A379K G380N L381Y insG G423A	F19S L72S, frameshift and L117stop	A29E T30S	Yes	Gene *bla*_VIM-2_ found

Notes: isolates from the PATRIC database (n=15) are designated by the PATRIC prefix, the clinical isolates from our own collection (n=10) — by the Pa prefix; R — resistant isolates; I — isolates with an intermediate level of resistance; S — susceptible isolates (according to CLSI criteria); frameshift — shift of the reading frame; stop — replacement of the coding codon with a stop codon, leading to the termination of protein synthesis; insS — serine insertion; insG — glycine insertion; WT — wild-type porin, in which there are no amino acid substitutions compared to the reference porin; carbapenemase genes are highlighted in bold (according to ResFinder).

Isolated substitutions (1–8 AAS) predominated in porins OpdD and OpdP, and this pattern of AAS was observed in both carbapenem-sensitive and carbapenem-insensitive isolates. Deletion of one nucleotide in the *opdD* gene, leading to premature termination of porin synthesis, was detected in 4 carbapenem-insensitive isolates (in 3 — resistant to meropenem and imipenem, and in 1 — with an intermediate level of resistance to meropenem). In 1 isolate resistant to meropenem and imipenem, the loss of functional porin OpdP resulted from the appearance of a stop codon in the nucleotide sequence encoding for position 81 of the protein.

## Discussion

In most cases, *P. aeruginosa* clinical isolates exhibit resistance to beta-lactam antibiotics, including carbapenems [22, 23]. Methods for detecting beta-lactamase-mediated resistance are quite simple and well implemented in the routine laboratory practice [24, 25]. At the same time, the analysis of chromosomal determinants of resistance is associated with difficulties with data processing. The proposed computer program is aimed at identifying the genetic determinants of non-plasmid antibiotic resistance and mutations in the *oprD*, *opdD*, *and opdP* genes of porins that transport carbapenems into the bacterial cell. This program also allows us to estimate changes in the amino acid structure of the respective proteins associated with resistance to carbapenem.

The analysis of 35 genomes (25 genomes of *P. aeruginosa* and 10 genomes of five other bacterial species) provided evidence for the usefulness of the proposed program in the search for *oprD*, *opdD*, and *opdP* genes and their mutations. The presence of these genes in all studied isolates of *P. aeruginosa* and their absence in the genomes of *A. baumannii*, *K. pneumoniae*, *E. coli*, *S. aureus*, and *E. faecium* indicates the specificity of the developed program in a search for target nucleotide sequences. Among *P. aeruginosa* isolates, the most diverse changes were found in the *oprD* gene, which corroborated with reports on the frequent occurrence of this gene in the carbapenem-resistant phenotype in *P. aeruginosa* [26].

The proposed program allowed us to identify the entire spectrum of theoretically possible types of mutations, including mutations leading to nonsynonymous amino acid substitutions, nonsense mutations (leading to the appearance of stop codons), insertions, deletions, and frameshifts.

The detection of carbapenem-resistant isolates containing the wild-type OprD can be due to the existence of alternative resistance mechanisms and, first of all, carbapenemases of the OXA groups (see Table 3). In addition, overexpression of the efflux system genes and mutations in genes of penicillin-binding proteins cannot be ruled out [23]. Further studies on molecular mechanisms of antibiotic resistance in bacteria are needed to provide a more comprehensive insight into the genetic basis of the resistant phenotypes.

Interestingly, different types of mutations, as well as their different numbers, were found in the *oprD*, *opdD*, and *opdP* genes. At the present stage, we have no tangible explanation for this intriguing statistical result. A mechanistic explanation of this phenomenon requires the knowledge of DNA repair in the genomes of *P. aeruginosa*, which is not available today.

Several strains, although resistant to imipenem, were found susceptible to meropenem. To explain this observation, we refer to the ability of meropenem to enter the cell via several alternative routes mediated by porins OprD, OpdD, or OpdP. For instance, if the *oprD* gene is mutated, meropenem can be transported via the non-mutated OpdD and OpdP porins. By contrast, imipenem has only one known “gateway” — porin OprD, which explains the discrepancy between imipenem and meropenem regarding the drug-susceptibility in the studied isolates of *P. aeruginosa*.

The proposed software product may have limitations associated with errors in sequencing and/or genome assembly, in which case the program produces the result “Gene not found”.

Overall, the developed algorithm allows for simultaneous search for resistance determinants in whole-genome sequences, the number of which is limited only by the abilities of the computer used.

## Conclusion

The proposed novel computer program is an effective tool for deciphering the molecular genetic mechanisms of bacterial (chromosomal) resistance to antibiotics. Its use made it possible to detect differences between the occurrences of mutations significant for resistance to carbapenems in the *oprD*, *opdD*, and *opdP* genes. The *oprD* genes were found to be more prone to mutations than the *opdP* genes. Due to the shunting of the porin-mediated transport of meropenem, conclusions about the resistance of a given strain to this antibiotic should be based not only on the detection of porin OprD abnormalities but also on the presence of significant mutations in the *opdP* and *opdD* genes. The present data suggest that meropenem is a more promising antibiotic than imipenem in the fight against *P. aeruginosa* strains with carbapenem resistance associated with impaired porin function.
